# Experiences of guided Internet-based cognitive-behavioural treatment for depression: A qualitative study

**DOI:** 10.1186/1471-244X-11-107

**Published:** 2011-06-30

**Authors:** Nina Bendelin, Hugo Hesser, Johan Dahl, Per Carlbring, Karin Zetterqvist Nelson, Gerhard Andersson

**Affiliations:** 1 Department of Behavioural Sciences and Learning, Swedish Institute for Disability Research, Linköping University, Linköping, Sweden; 2 Department of Psychology, Umeå University, Umeå, Sweden; 3 Department of Clinical Neuroscience, Psychiatry Section, Karolinska Institutet, Stockholm, Sweden

**Keywords:** Internet treatment, depression, cognitive behaviour therapy, self-help

## Abstract

**Background:**

Internet-based self-help treatment with minimal therapist contact has been shown to have an effect in treating various conditions. The objective of this study was to explore participants' views of Internet administrated guided self-help treatment for depression.

**Methods:**

In-depth interviews were conducted with 12 strategically selected participants and qualitative methods with components of both thematic analysis and grounded theory were used in the analyses.

**Results:**

Three distinct change processes relating to how participants worked with the treatment material emerged which were categorized as (a) Readers, (b) Strivers, and (c) Doers. These processes dealt with attitudes towards treatment, views on motivational aspects of the treatment, and perceptions of consequences of the treatment.

**Conclusions:**

We conclude that the findings correspond with existing theoretical models of face-to-face psychotherapy within qualitative process research. Persons who take responsibility for the treatment and also attribute success to themselves appear to benefit more. Motivation is a crucial aspect of guided self-help in the treatment of depression.

## Background

The Internet offers a new way of providing psychological treatment for common mental health problems [1]. Internet treatment has been described as a treatment that is operationalized and transformed for delivery via the Internet [2]. They are usually highly structured, and self-guided or partly self-guided with an identified therapist [3]. Internet interventions may however also resemble traditional psychotherapy with scheduled sessions [4], but overall a characteristic feature is that that patients are reached from a distance and that therapist time is reduced compared with face-to-face treatment [5]. Several independent research groups have developed Internet-based cognitive-behavioral interventions [6,7], and randomized controlled trials suggest that Internet-based treatment with minimal therapist contact via e-mail can be effective in treating various conditions [8], including depression [9] with moderate to large effect sizes. Although several studies have shown that guided Internet treatment of depression [10] can lead to reduced symptoms, less is known about how participants experience these treatments. Reviews have however been conducted on the reasons why people fail to adhere to computerized interventions [11,12]. A few qualitative studies have investigated experiences of guided self-help [13], including computerized treatments [14], showing that both advantages and disadvantages are reported by patients who have received guided self-help for depression. One study investigated experiences of an Internet depression treatment that was delivered in real time with scheduled online session appointments [15]. Results revealed two themes: developing a virtual relationship with a therapis and the process of communicating thoughts and emotions via an online medium. There is also a recent study on unguided Internet-delivered depression treatment [16] which concluded that the program tested did not work and that provision of support could improve outcomes.

Studies using qualitative methods can be helpful when investigating the potential benefits and disadvantages of treatment and explorative methodology of participants' experience can help us understand the mechanism of change. Since relatively little is known regarding predictors and mediators of change in Internet treatments [17], qualitative research may be justified help identify the unique aspects of Internet treatment. To our knowledge, no study has to date investigated how participants experience guided Internet-based self-help treatment for depression. This study therefore used qualitative methods to explore participants' experience of an Internet administrated guided self-help treatment for depression. We conducted the study alongside a randomized controlled trial [18], in which we investigated two forms of Internet treatment for major depression: e-mail therapy and guided self-help [19]. The overall aim of the study was to obtain a detailed understanding of participants' experience of the treatment that could contribute to improve the effectiveness and reduce dropouts in Internet-based depression treatments.

## Methods

### Participants

Participants were recruited from the randomized controlled trial that compared two forms of Internet-based cognitive-behavioural therapy for depression [19]. In that study 88 patients with mild to moderate depression were recruited via advertisement and later randomized to Internet-based self-help with minimal therapist contact (self-help group), individualised Internet-based email-therapy (email group), or to a delayed treatment waiting-list condition (control group). The first group received an Internet-administrated self-help treatment that consisted of 114 pages of text divided into seven modules over a period of 8 weeks. The second group received purely e-mail based treatment with no prepared text, but in principle based on the same procedures and cognitive-behavioural therapy (CBT). The control group later received the Internet-based guided self-help, but with less therapist contact (i.e., a few minutes per week, responding to questions sent by the participant) and participants from this group were also included in this study. More detailed information about the study is presented in the treatment trial [19].

Six months after treatment participants were contacted by phone and invited to participate in a longer interview concerning their experiences of the treatment they had received. This was done in association with a follow-up and only persons participating in the follow-up were asked for participation in this study. Of the 74 that were contacted, 48 (64%) expressed an interest in taking part in the study. Twelve of the 48 interested were selected and consented to participate in the interview. The 36% who declined taking part of the study did not differ systematically from the larger group (in terms of depression scores on the Beck Depression Inventory [20]), and the main reason was lack of time. A purposive sample selection according to the maximum variation strategy was used [21], based on both treatment received and overall improvement. The purpose of this selection process was to explore the variation within participants, to ensure diversity in opinion, as well as the shared experiences of the participants who had received Internet-based treatment for depression. Overall improvement was measured with a revised version of Clinical Global Impressions-Improvement Scale (CGI-I) [22] at 6-month follow-up compared to pre-treatment functioning. The CGI-I measures overall improvement on a 4 grade scale: "very much improved", "much improved", "minimally improved" or "no change". The latter category also covers deterioration. Interviewer was blind to treatment status (e.g., guided self-help vs. e-mail treatment).

The primary aim of the strategic sample selection was to include the same number of participants in relation to their treatment group and their overall improvement. Only one participant whose improvement was graded as "no change" wanted to participate in the study. This was however representative for the treatment trial where only 5.9% showed no change on the CGI-I. In order to obtain a better understanding of experiences of those who had not made large improvements, two more participants whose improvements were graded as "minimally improved" were interviewed. Table 1 presents the 12 selected participants demographics, treatment group and improvement as measured with CGI-I.

**Table 1:** Characteristics of selected participants

Participant	Gender	Age	Martial status	**Employment**^**a**^	**Treatment group**^**b**^	**CGI-I**^**c**^
1	Female	62	Single	Employed	Email	Very much
2	Male	40	Married	Employed	Email	Much
3	Male	61	Married	Registered sick	Email	Minimally
4	Male	30	Partner	Employed	Email	No change
5	Female	29	Partner	Employed	Self-help	Very much
6	Female	28	Partner	Employed	Self-help	Much
7	Male	22	Partner	Student	Self-help	Minimally
8	Male	33	Single	Employed	Self-help	Minimally
9	Male	20	Single	Student	Control	Very much
10	Female	60	Single	Registered sick	Control	Much
11	Female	47	Single	Employed	Control	Minimally
12	Female	24	Single	Employed	Control	Minimally

^a^Employed; full time or part time

^b^The control group completed the self-help program after the active treatment groups had finished their treatment

^c^Improvement measured with CGI-I, Clinical Global Impressions-Improvement, at 6-month follow-up

Four participants from each treatment group were interviewed. Of the 12 participants whose interviews were analysed, 6 were female. Age ranged from 20 to 62 years with a mean age of 36.3 years (*SD *= 16.5 years). All were native Swedes and had an educational level of collage/university or higher. Seven participants were single. Eight had employment, 2 were on sick-leave and 2 were students. Three participants had previously received psychological treatment. Only 1 participant was diagnosed with another diagnosis (simple phobia) in addition to major depression disorder.

Before the interview information about the overall purpose of the study was given and participants gave informed consent. The medical ethics committee in Linköping, Sweden, approved the protocol as part of the larger treatment study [19].

### Material and Procedure

The twelve selected participants were interviewed. All interviews were face-to-face and held within eight to ten months after the treatment had ended. Two undergraduate M.Sc. clinical psychology students conducted the interview at the university clinic. The interviewers had not been part of the treatment study or had any previous contact with the participants. The interviews ranged from 42 to 111 minutes in length, with a mean of 68 minutes. All interviews were audio taped and transcribed. In order to secure the confidentiality of the participants, we made minor alterations to prevent identification of cases (e.g., names and places that could identify the participant).

The interview was based on the Client Change Interview [23], and the primary aim was to assess aspects of clients' experiences of the treatment. The interview consisted of open-ended questions, which were used as a guide to explore the clients' experiences of change and their understanding of what factors might have helped during treatment. Questions in the interview guide were classified in the following subject headings: (a) Clients general impression of the treatment, (b) how the clients perceived the way treatment was conducted, and (c) changes perceived by clients over the course of the treatment and their understanding of what might have brought these changes about.

### Analysis

**Figure 1:**
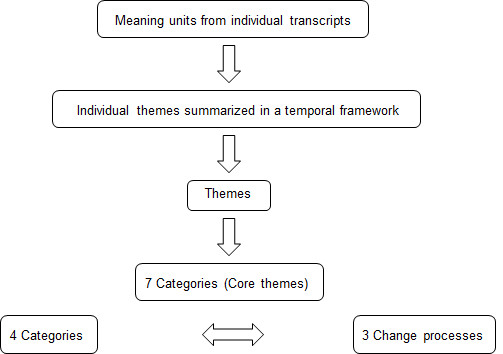
**Flow chart of the stages in the analysis used in the study**.

A method comprising components from both thematic analysis [24] and grounded theory [25] was used in the analysis of the material. While these two approaches are related thematic analysis seek to summarise/encapsulate the data, but not necessarily with the aim of developing a theory to explain it in the same sense [26]. We wanted to expand a bit from a pure thematic analysis but did not expect to reach saturation and reach a new theory which are goals in grounded theory [27]. A flow chart of the analysis used in the current study is shown in Figure 1. In order to optimize the analytic process the researchers first coded each other's transcripts separately without referring to other interviews. During the first stage of the analysis the two interviewers worked independently. They coded each other's transcripts into meaningful units. The units represented statements that the participant made that conveyed a meaningful concept. After coding independently, the researchers then compared the meaning units in an iterative process and discussed emerging themes from individual transcripts among all the transcripts. The meaning units were analysed in order to obtain individual themes, which remained close to the data and represented the particular participant's experience. For each participant the individual theme was summarized in a temporal framework of change as a way of clarifying change processes [23]. During the next stage of the analysis process commonalities among the individual themes were analysed and new themes emerged. The new themes were then categorized. Themes that shared similarities were grouped into a category. Seven categories were obtained. In order to remain close to the data, the transcripts and the individual themes were then re-examined based on the seven new categories that had emerged. During the reanalysis of the material the seven categories were reduced to four and communalities of experiences based on the four categories formed three distinct groups among participants. Three different change processes among participants' experiences in terms of the four categories, characterized the three groups. Keywords that were significant for the three change processes were formed from themes among participants in the groups. The four categories and the three change processes are presented in the result section.

To ensure the interpretation of the data the researchers conducted a number of credibility checks. The researchers constantly checked individual interpretation and ensured that they reached consensus. They also reanalysed material specifically looking for discrepancy between the material and the result. Throughout the analysis the two researchers consulted with experienced researchers in the field of Internet-based self-help treatment and qualitative research to discuss the result of the analysis. Two of the more experienced researchers had been part of the intial study and had extensive experience from conducting trials on Internet treatment. One researcher with expertise in qualitative research had not previously worked with Internet treatment research and supervised the M.Sc. students independently. Examples of data are provided to enable the reader to judge the fit between the data and the interpretation of them.

## Results

### Overall results

Four categories (core themes) emerged from the analysis of participants' accounts of the treatment. Two of the four categories comprised themes in relation to how the participants worked with the treatment material and experiences of how the treatment accomplished motivating participants: Working Process and Motivation. The two other categories covered themes of participants' opinions of the treatment and what skills and knowledge they had obtained from the treatment: Attitudes towards treatment and Consequences of treatment. The four categories were evident within all participants' accounts. Within the four categories, three groups of experiences regarding different change processes emerged: (a) *Readers*, (b) *Strivers*, and (c) *Doers*. Keywords that corresponded with the change processes were formed from participants' themes within each category (see Table 2). The three change processes are illustrated within the four categories in the following text and quotations in Table 3.

**Table 2:** Participants themes presented as keywords, change processes and categories

Category	Keywords	Change process	Themes	Participant nr
	Reading	*Readers*	Read	4, 8, 11
	Giving up		Eye-opener	4, 8
			Sporadic work	4, 8
			Giving up/avoid	8, 11, 12
**Working Process**	Trying to practice	*Strivers*	Interpreting	2, 3, 7, 12
			Ambivalent regarding practice	2, 7, 12
			Uncertain	2, 3, 7, 12
			Trying to redefine	2, 3, 12
	Practice	*Doers*	Methodical work	1, 5, 6, 9, 10
	Apply insights		Emotional insights	1, 5, 6, 9, 10
			Apply/practice	1, 5, 6, 9, 10
			Redefine barriers	1, 5, 6, 9, 10
	Lack of support	*Readers*	Lack of support	4, 8, 11
			Not seen	8, 11
			No response	8
**Motivation**	Insufficient support	*Strivers*	Missing conversation	2, 3, 12
			High demands	2, 7, 12
			Need of more support	2, 3, 12
	Proximal support	*Doers*	Self-reinforcement	1, 5, 6, 9,10
			Optimal support	5, 6, 9, 10
			Autonomy	5, 6, 9, 10
			Security	1, 5, 6, 9, 10
	Disappointed	*Readers*	Not suited for me	4, 8, 12, 11
			Ambivalence	4, 12
**Attitudes towards treatment**	Uncertain	*Strivers*	Avoidance	7, 12
			Skepticism/fear	2, 3, 7
	Helpful	*Doers*	Works	1, 5, 6, 9, 10
			Faith in program	1, 5, 6, 9, 10
	Awareness	*Readers*	Disappointed/shameful	4, 8, 11
			Lonely	4, 8, 11
			Lack of reinforcement	8, 11
			Awareness of needs	4, 8
**Consequences of treatment**	Integrated insights	*Strivers*	Ambivalence	2, 7
			Dependence	12
			Needed more help	3, 7, 12
			Gained insights	2, 3, 7
	Self-sufficiency	*Doers*	Emotional insights	1, 5, 6, 9, 10
			Generalization	5, 6, 9, 10
			Model of treatment	1, 6, 9, 10
			Acceptance	1, 5, 6, 9,10

**Table 3:** Quotes from participants

Q 1 '...you got some information about a few things, statements, that you recognized and was relevant to you...I guess it (the treatment program) gave me something...The material you read, it made you, it started something. Something new' (Participant 8).
Q 2 '.. I think I've realized that I'm kind of, I realized that I'm kind of lazy, by nature, one likes to take shortcuts, and perhaps not do the things you really ought to do. It might feel difficult or you can easily put these must-remember thoughts aside. I have realized that it's really easy to do that. If you got an assignment that made it clear that one should do so and so, then it might feel difficult, it was interesting though...but still, it felt difficult ' (Participant 8).
Q 3 'Yes, I got rid of that thing (laughter). Well not really, I stopped doing all those assignments....it was kind of a relief when it was over' (Participant 4).
Q 4 '...you tried to register what you did at every moment. What you thought and how you felt. And I tried to do it regularly during the day, both at work and home.... When you write it down, it forces you to put your thoughts into words. And there's something positive about that, when you write it down, it's almost like doing it, and then you can go back and look at it' (Participant 2).
Q 5 'I sometimes thought it was hard, that it was tough...I suppose, if it was a good day I got it (treatment assignments) done (laughter)' (Participant 12).
Q 6 'When you had read something, for example the night before, then you tried to practice it the following day, and you put effort into it. For example every little action that you did, you try to think why am I doing this...And it was a lot of work, until you could practice it on a more unconscious level' (Participant 9).
Q 7 'I sometimes feel it would be good for me to do the week (with behavioural activation) again' (Participant 10).
Q 8 'Yes, I guess it was that week (with behavioural activation), after all, I think, because it was tough, and it was noticeable, made me so aware...' (Participant 10).
Q 9 'Yes, it's important for me that I'm able to show someone what I've accomplished, and get some kind of response, someone who expresses his or her opinion. I guess, you get some kind of assurance or something like that. That's not what happened here' (Participant 8).
Q 10 'I felt guilty because I didn't do all the assignments (laughter). Yes, it became an extra burden, so I felt like, this is not what I really need' (Participant 4).
Q 11 'Put simple, I couldn't find time for it (the treatment program)' (Participant 4).
Q 12 '...someone to talk to, I think that would have motivated me' (Participant 12).
Q 13 'I felt like I couldn't control it, it became something of a burden that was added to me, you could say that it created anxiety, one more thing that I couldn't do or complete, that I promised to do' (Participant 2).
Q 14 'Of course, much of what I did, I did because I promised to do it (the treatment program) and then I felt like I had to do it' (Participant 2).
Q 15 'I think it was important for my self-esteem. To feel that I did it on my own, like I was able to do things' (Participant 6).
Q 16 '... if you needed to consult someone about something there was someone there. If I needed, I could choose to take contact' (Participant 5).
Q 17 'You felt like, I feel fine now, why should I continue (with the treatment)' (Participant 9).
Q 18 'The expectations were probably higher than the outcome' (Participant 8).
Q 19 'Although it wasn't what I expected, or whished for, but that had a lot to do with me....I still believe it's a concept that works' (Participant 4).
Q 20 'Some part of me was all the time scared like, to work with it, I don't know...I don't think it was the material that frightened me...What if it goes out of control?' (Participant 7).
Q 21 'I don't believe in going to a "shrink", like the Americans do, right, I don't believe in that. But...if you feel bad, and if I'd find myself in some kind of crisis or something, right. Then I believe, or I know, that this is a good model to help you through a crisis. When a depression appears I know that this is a good model' (Participant 1).
Q 22 'I sometimes find it difficult to sit down and talk to someone, who'd try to understand me, and this way I got to work with myself...in a completely different way' (Participant 5).
Q 23 '...perhaps, it has affected me, without my knowledge. But, I can't think on any specific part, that I'm thinking about, that I'm using' (Participant 4).
Q 24 'I gave up after a while and I guess I thought: There's no purpose of me sitting here doing this, it's not going to make a difference anyway' (Participant 11).
Q 25 '..And in that way I've become more aware of how strange I sometimes think and how that affects my mood and I've become better on thinking other thoughts' (Participant 12).
Q 26 'Actually, the treatment program could have made a larger impact than it did, but I guess that's because I was too scared to work with it, I didn't use the material enough...' (Participant 7).
Q 27 'Perhaps I should look at it (the treatment material) some more... At least it's not uninteresting, it's educational....I kind of see it as a course, a course that you should look at some more, because when you think of it, it wasn't that bad' (Participant 7).
Q 28 'It feels like I've regained the ability to take control of my life' (Participant 10).
'It (the treatment program) has meant a great, great deal to me in several different ways. Personally I feel a lot better, and then also, at work too, I work in, as a nurse ... So I used it both in my work and on my self' (Participant 1).
Q 29 'I don't feel that it's overwhelming, it's much and it's though, but you always know that you're going to get through it' (Participant 9).
Q 30 'That's why I now look at depression and feeling down as a natural thing, which happens to people...' (Participant 9).

### Working Process

#### Readers

Three participants identified gained awareness through reading the material as a main theme in their accounts of how they worked with the treatment material. However, they were not able to or did not want to put their newfound insights into practice, nor did they report that they applied treatment strategies in everyday life. Instead, they expressed that they had required a general theoretical understanding of treatment principals and/or themselves by reading the material (Q1). Working sporadically with the material presented to them throughout the course of the treatment, either going through it too fast or skipping parts of it, also seemed to be a significant theme within "Readers" narratives (Q 2). Determining workload and work pace on their own appeared to be an obstacle and was often the reason why participants did not complete modules or dropped out of treatment altogether. Deciding not to continue working with the material seemed to function as a way of handling difficulties (Q 3).

#### Strivers

In contrast to the descriptions made by "Readers", four participants appeared to express that they not only read but also worked with the material in a practical way. They reported that they completed treatment assignments and were therefore able to give specific details of their working process to substantiate their perception of the treatment (Q 4). Although their approach to work in terms of completing home-work assignments to some extent had made it possible for them to integrate treatment in everyday life, they also expressed ambivalence, and sometimes scepticism, regarding practicing insights and working on their own. One female participant described her way of working in treatment, which to a large extent signified the working process of "Strivers" (Q 5). This ambivalence towards practice-oriented work appeared to play an important role in their experience regarding not being able to profit from the treatment to a full extent.

#### Doers

Testing the material, applying it and putting insights into practice appeared to be themes for "Doers" within their accounts of how they worked with the material. When the participants talked about their working process, they gave specific examples of key moments in treatment and reported that they worked with the material in a practical way, applying it to experiences and real-life events (Q 6) A structured and a methodical way of working appeared to be a significant theme within participants' narratives in this group. If and when barriers occurred their practice-oriented approach seemed to have helped them overcome obstacles (Q 7). Several participants also expressed that facing difficulties and confronting challenges in treatment were of great significance, and often described as the most valuable lesson they had received (Q 8).

### Motivation

#### Readers

Lack of support, missing someone to talk to in real life, and need for a push to continue when it became difficult, were emphasized among some participants and explicitly outlined by the "Readers" (Q 9). Some even expressed that the treatment program was added as an additional burden (Q 10). Lack of time was frequently described as a reason for declining motivation to continue treatment (Q 11).

#### Strivers

Inadequate support in relation to needs and a wish for more contact with the therapists was stressed by four participants as a main reason for not feeling motivated. Although they felt that they had support, they stated a wish for more contact in form of conversation in order to get a more profound understanding of their problem and/or to help them overcome barriers in treatment (Q 12). Some reported feeling stressed by too high expectations and demands from the therapist behind the program and reported that as a factor contributing to loss of motivation (Q 13). Rather than being motivated by the treatment, several appeared to be driven by a sense of duty (Q 14).

#### Doers

As a facilitating factor for motivation several participants emphasized a proximal degree of support from the treatment program. They stressed the importance of working on their own and appreciated the responsibility that came with the program (Q 15). At the same time, if faced with a difficult situation they were not able to handle, they found that they had a security backup system in the treatment program (Q 16).

However, some reported improvement as cause for lowering motivation to continue with the treatment (Q 17).

### Attitudes towards treatment

#### Readers

Disappointment in relation to high expectations was a significant theme for "Readers" within their accounts of attitudes towards the treatment (Q 18).

However, even when they reported that the program for different reasons was not suited for them, they expressed that the treatment might benefit others (Q 19).

Failing to improve from treatment made participants express a wish of going back and working more with the material hopping to profit from it.

#### Strivers

Themes regarding doubt and uncertainty emerged within "Strivers" accounts of attitudes towards the treatment. They expressed scepticism about cognitive-behavioural therapy or Internet self-help treatment. Some found it scary to initiate contact with an unknown person over the Internet. Other expressed a fear because treatment made it possible for them to explore and work with their depression (Q 20).

#### Doers

Themes regarding the usefulness and helpfulness of Internet self-help treatment emerged in the "Doers" accounts of their attitudes towards the treatment. In comparison to traditional remedies participants reported treatment as a new, exciting and better way of receiving care (Q 21). Several participants appreciated the independence and that the program enabled them to work on their own. Some acknowledged that they did not have to meet a therapist face-to-face (Q 22).

### Consequences of treatment

#### Readers

No change of their situation, not having gained adequate skills to deal with the depression, and a wish for more help, were identified as main themes in three participants' accounts. Although they expressed that they had obtained general insights into their problems and had become more aware of their needs as a consequence of treatment, they had not received skills to help them deal with their depression (Q 23).

The lack of treatment effect had made them feel lonely, shameful and disappointed (Q 24). As a result of not being able to profit from the treatment they often reported a wish for more help.

#### Strivers

Four participants emphasised that the treatment had encouraged them to revise their perceptions of depression and of themselves. They seemed to have gained a greater understanding of themselves and their current situation by working with the material and expressed that they had acquired specific insights to help them cope with their depression (Q 25). However, ambivalence regarding treatment effect also appeared to be a main theme within their narratives. They often expressed concern whether they could have improved more if they had worked differently with the material or had received another treatment (Q 26). This ambivalence was also expressed as dependence towards treatment and as a wish to receive more therapy in hope to get better (Q 27).

#### Doers

Five participants underlined their autonomy and self-sufficiency in their accounts of consequences of the treatment. Participants in the doers group had not only received a greater understanding of their particular problems but were also able to practice skills, insights and approaches to problem solving in everyday life. They appeared to have acquired a model of the treatment that enabled them to develop skills and apply them in various settings (Q 28). The fact that they had accomplished "beating" the depression on their own had made them believe in their own ability to cope with eventual relapses (Q 29). They also expressed acceptance of periods of feeling depressed and of themselves in their statements regarding consequences of treatment (Q 30).

## Discussion

The aim of this study was to explore participants' experience of Internet administrated treatment for depression using qualitative methods. The analysis of participants' accounts of the treatment sixth months after the treatment yielded three distinct patterns of change. These related to participants' motivational experience of the treatment, how they worked with the treatment material, their attitudes towards the treatment, and their perception of what skills and knowledge they had gained from the treatment.

Overall, the results correspond with existing theoretical models of change in traditional face-to face psychotherapy for depression within the qualitative process research [28,29]. The three change processes are partly in line with the three patterns of change described in study of clients' experience of a face-to-face short-term counselling [30]. The current study also indicates that therapeutic work in an Internet-based treatment is as a dynamic process, and that the treatment is perceived differently depending on expectations and outcome. This is in line with a previous qualitative study on guided self-help [13], and another qualitative study on how a mental health self-help clinic was perceived [31]

Although more research is needed to draw definitive conclusions, this study offers some clinical implications of value to those developing Internet-based self-help treatments. The results indicate that participants who reported that they only worked sporadically with the treatment, alternatively that they did not want to or could not work practically with the treatment material, were less inclined to view the treatment positively and also reported a less favourable outcome of the treatment. In comparison, participants who had a practical "hands-on" approach to work (testing and applying treatment strategies), expressed that they preferred the treatment format and had integrated the treatment principles in their everyday life. Redefining obstacles in treatment, confronting challenges, believing in their own ability, and acceptance of depression and of themselves also seemed to be significant themes within the narratives of participants who expressed improvement and a favourable outcome. Those themes seemed to be consistent with the findings in previous research on successful therapeutic work [28,29].

Participants also gave different accounts in relation to the three change processes of how they perceived the support and the motivational aspects of the treatment. Some appreciated that they had to work on their own and felt that they had a "back up" support system, while others missed real-life conversation or felt pressured by the treatment program. This is partly in line with previous research which showed that an intense relationship with the therapist resulted in interrupted change patterns in time-limited counselling [30]. Our results indicate that both too much contact with the therapists (in particular if it is perceived as inadequate) and a lack of contact can both be hindering factors for treatment success. While participants may need some form of support in Internet administrated treatments [32], the current study also points to the importance of autonomy and feelings of self-efficacy in successful therapeutic work. In line with previous research [33], the findings highlight that an Internet program should take into account the non-specific factors of therapeutic work by developing a sufficient amount of support [9]. But in doing so, one should not jump to the conclusion that much more support will necessarily lead to better outcome. It might be that too much contact from the treatment program render loss in self-efficacy and make the participant believe that he or she is not the agent in the treatment or alternatively that the loss of face-to-face contact becomes more apparent when the contact is via electronic media [15]. Indeed, some expressed that they missed conversation and needed additional support, which might indicate that contact (e.g. via e-mail or face-to-face) is important to a varying degree depending on the participant. Individuals who have the ability and motivation to work on their own and have a structured and practical approach to work, are perhaps more suited for this form of treatment than others. More research is needed to determine if this is the case. On the other hand, a program designed to fit participants' motivation and ability (e.g. giving participants more time to complete treatment or more e-mail contact) might be suitable to increase adherence [34]. Indeed, tailoring of treatment could be feasible in particular in depression as comorbidity with anxiety disorders and somatic disorders is common [35].

This was an exploratory study and the interpretations of the data should be considered within the context of qualitative research [36]. The results can not be regarded as representative for all people who receive Internet administrated treatment for depression. The sample was limited, small and selected, and from a qualitative research point of view a larger number of participants would have been preferred given the way we analyzed our data as a fuller description might have emereged. Most importantly, the study included only one participant who had not improved at all from treatment (as measured with the CGI-I). If more such participants had been included the results might have been different. However, on account of this misrepresentation two more participants whose improvements were graded as minimally change were interviewed. All of the participants had an educational level of collage or higher, which may also limit how well our findings can be generalized. While qualitative research often involves relatively few participants, our sample may have been too small. Moreover, because the interviews took place six months after treatment had ended, the results rely on participants' memories of treatment, which could of course be subject to bias and also dependent on how well the treatment worked. This might make it difficult to draw any firm conclusions about sequences of change during treatment, participants' experiences of treatment content or attitudes towards treatment, because clients tend to construct memories in accordance with their present situation [37].

Another limitiation of the study relates to the potential bias since two of the involved researchers had worked with the treatment trial. We tried to handle the bias by involving a researcher with no previous experience of Internet research and also included a fifth co-author to handle the possible bias. Moreover, we constantly reflected on the research process and our own role when coding and interpreting the interviews [38], and were careful ton include not only participants who were very pleased with the intervention but also persons who had not improved.

## Conclusions

The findings correspond with existing theoretical models of face-to-face psychotherapy within qualitative process research. Persons who take responsibility for the treatment and also attribute success to themselves appear to benefit more from Internet-based treatment. Further qualitative research is needed to investigate how participants experience Internet-based treatment for other problems than depression. This might not only cast light upon how Internet based self-help treatments work but may also give additional information about the active ingredients and the mechanism of change in face-to-face psychotherapy, in particular in relation to common and specific factors.

## Competing interests

The authors declare that they have no competing interests.

## Authors' contributions

NB, JD and GA conceived of the study and its design. HH and GA worked with the write-up and reanalyses, and KZN supervised the qualitative analyses, PC added input on the treatment delivered and all authors read, commented on and approved of the manuscript.

## Pre-publication history

The pre-publication history for this paper can be accessed here:

http://www.biomedcentral.com/1471-244X/11/107/prepub
